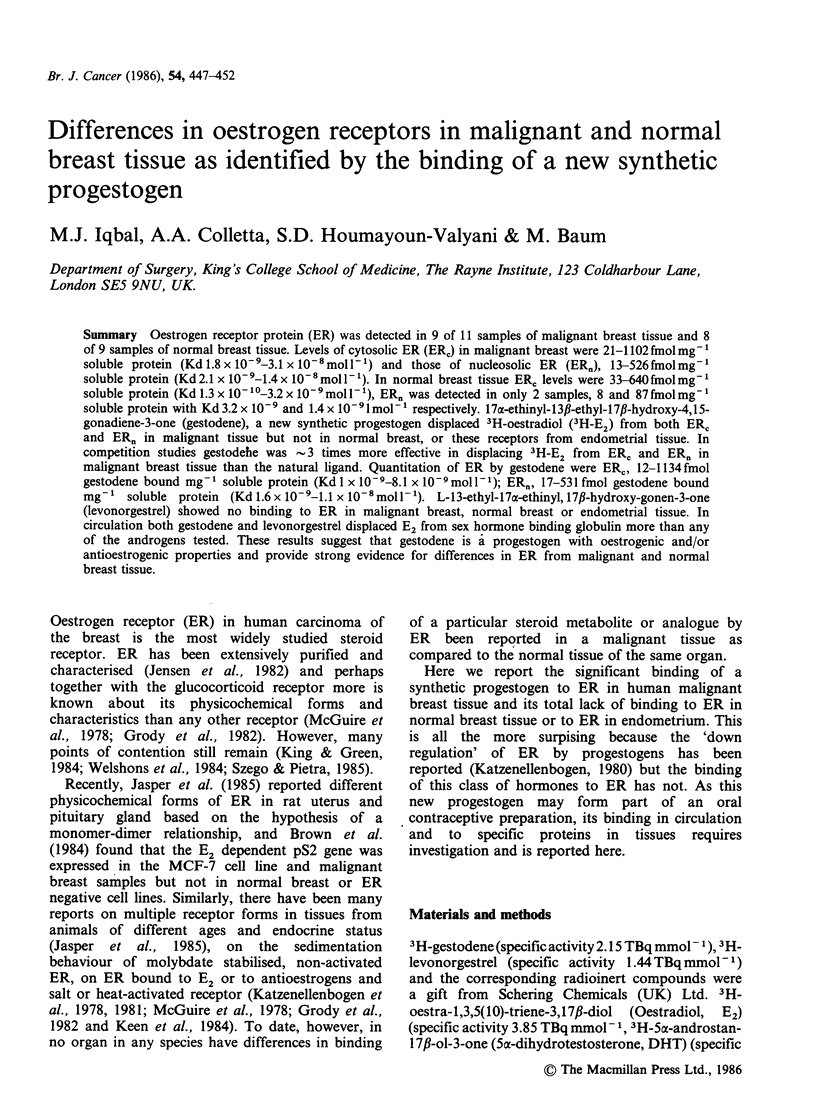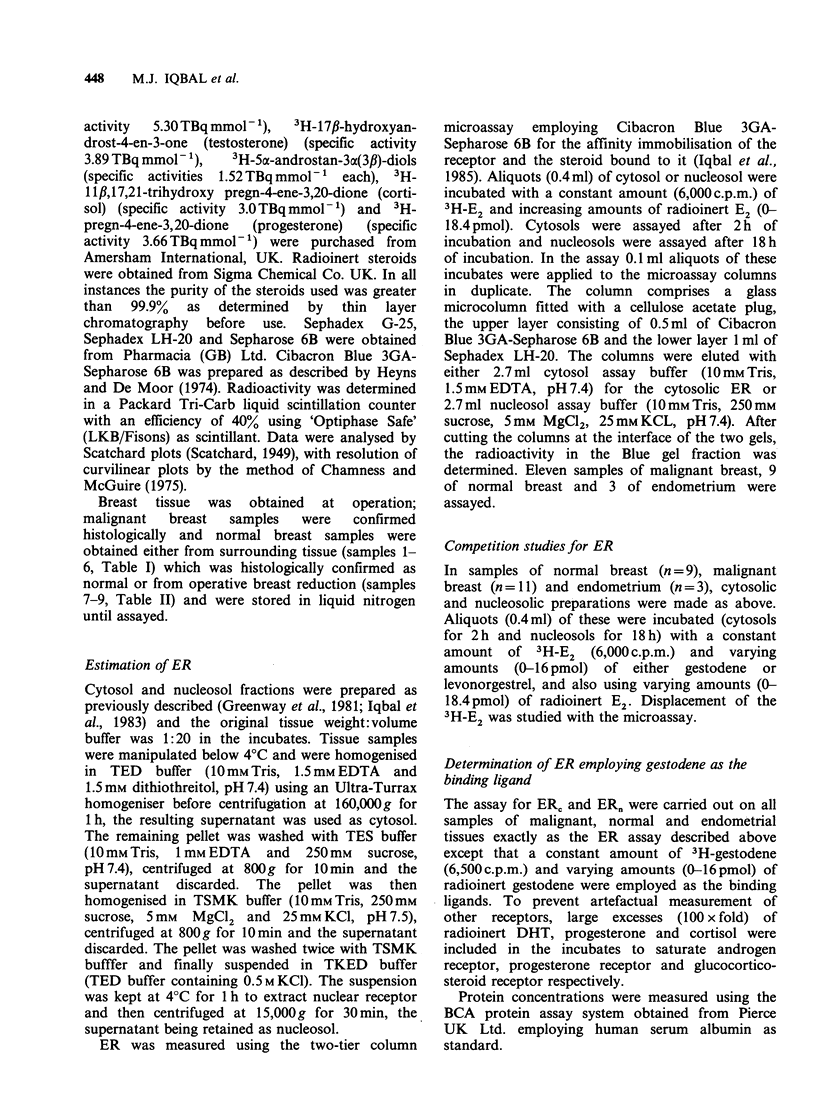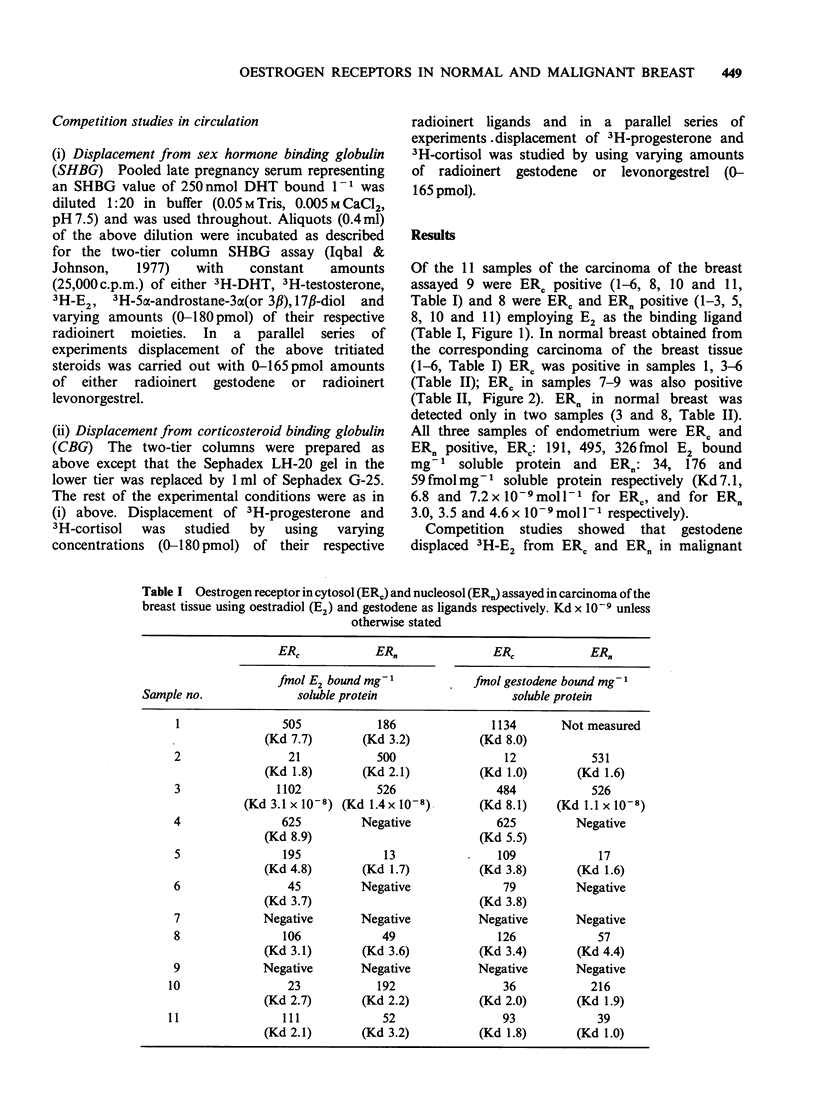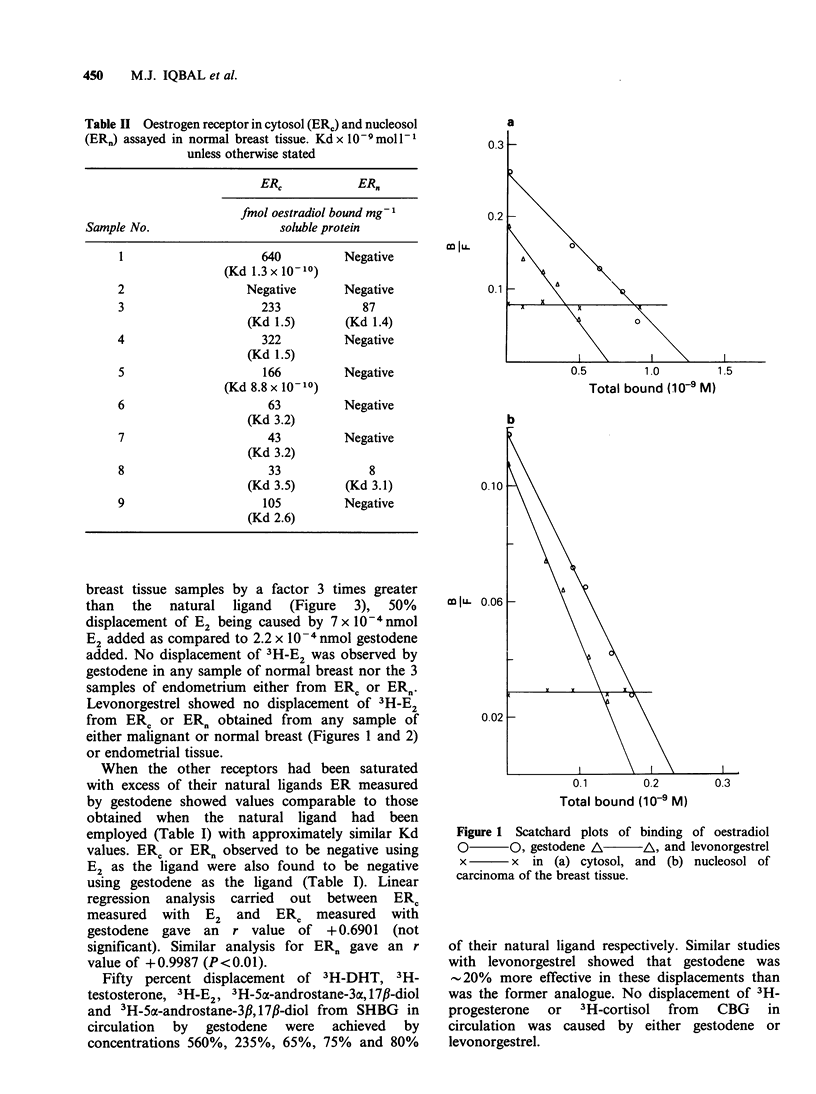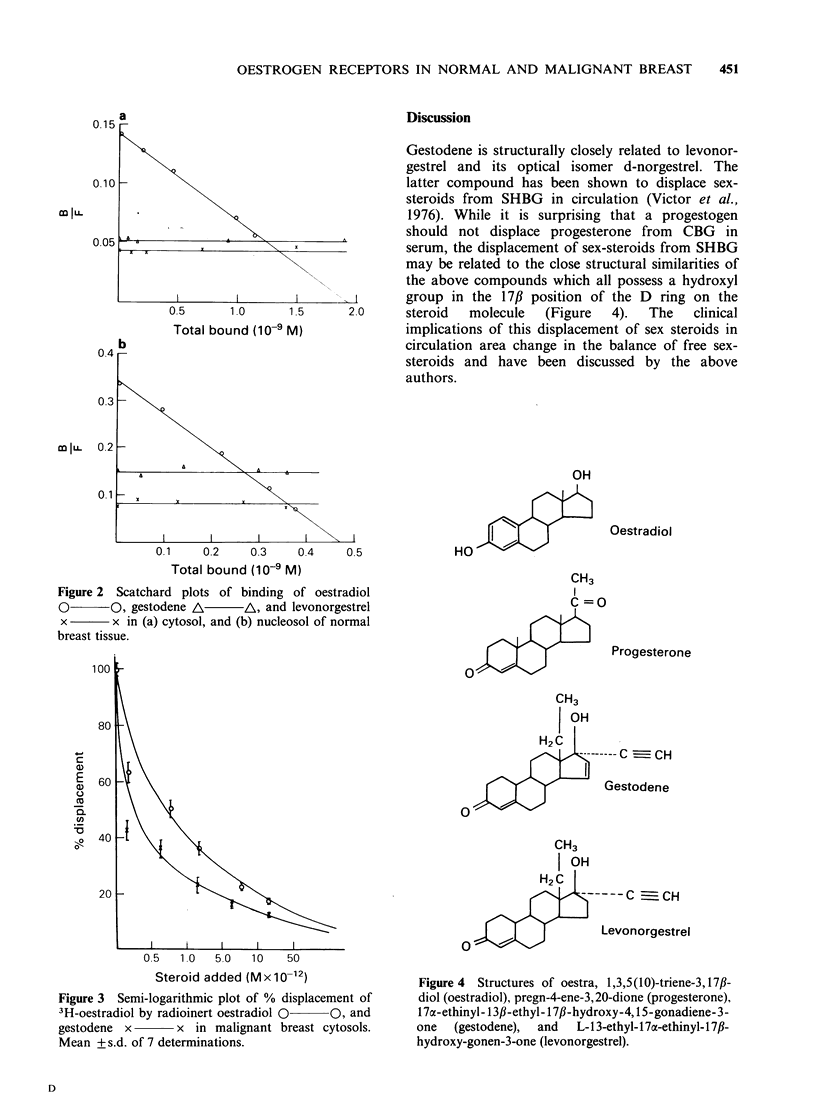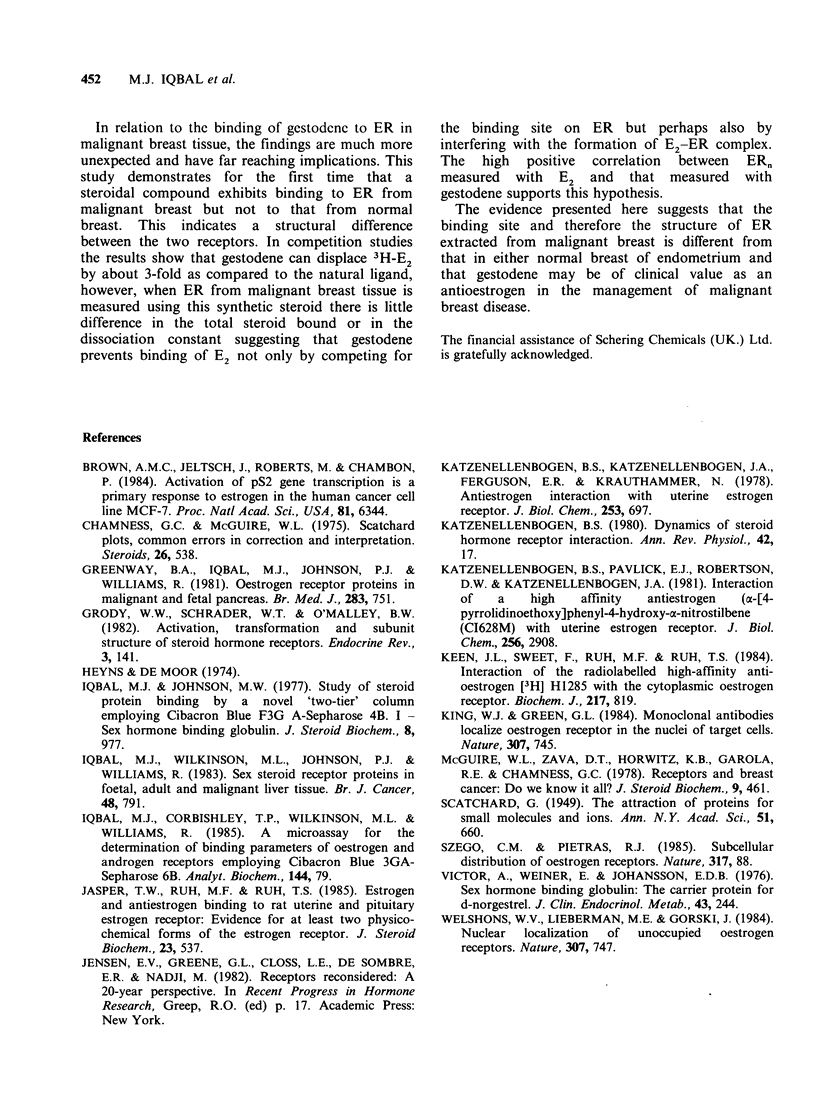# Differences in oestrogen receptors in malignant and normal breast tissue as identified by the binding of a new synthetic progestogen.

**DOI:** 10.1038/bjc.1986.196

**Published:** 1986-09

**Authors:** M. J. Iqbal, A. A. Colletta, S. D. Houmayoun-Valyani, M. Baum

## Abstract

Oestrogen receptor protein (ER) was detected in 9 of 11 samples of malignant breast tissue and 8 of 9 samples of normal breast tissue. Levels of cytosolic ER (ERc) in malignant breast were 21-1102 fmol mg-1 soluble protein (Kd 1.8 X 10(-9)-3.1 X 10(-8) mol l-1) and those of nucleosolic ER (ERn), 13-526 fmol mg-1 soluble protein (Kd 2.1 X 10(-9)-1.4 X 10(-8) mol l-1). In normal breast tissue ERc levels were 33-640 fmol mg-1 soluble protein (Kd 1.3 X 10(-10)-3.2 X 10(-9) mol l-1), ERn was detected in only 2 samples, 8 and 87 fmol mg-1 soluble protein with Kd 3.2 X 10(-9) and 1.4 X 10(-9) l mol-1 respectively. 17 alpha-ethinyl-13 beta-ethyl-17 beta-hydroxy-4,15-gonadiene-3-one (gestodene), a new synthetic progestogen displaced 3H-oestradiol (3H-E2) from both ERc and ERn in malignant tissue but not in normal breast, or these receptors from endometrial tissue. In competition studies gestodene was approximately 3 times more effective in displacing 3H-E2 from ERc and ERn in malignant breast tissue than the natural ligand. Quantitation of ER by gestodene were ERc, 12-1134 fmol gestodene bound mg-1 soluble protein (Kd 1 X 10(-9)-8.1 X 10(-9) mol l-1); ERn, 17-531 fmol gestodene bound mg-1 soluble protein (Kd 1.6 X 10(-9)-1.1 X 10(-8) mol l-1). L-13-ethyl-17 alpha-ethinyl, 17 beta-hydroxy-gonen-3-one (levonorgestrel) showed no binding to ER in malignant breast, normal breast or endometrial tissue. In circulation both gestodene and levonorgestrel displaced E2 from sex hormone binding globulin more than any of the androgens tested. These results suggest that gestodene is a progestogen with oestrogenic and/or antioestrogenic properties and provide strong evidence for differences in ER from malignant and normal breast tissue.


Br. J. Cancer (1986), **54**, 447–452

### Differences in oestrogen receptors in malignant and normal breast tissue as identified by the binding of a new synthetic progestogen

M.J. Iqbal, A.A. Colletta, S.D. Houmayoun-Valyani & M. Baum

*Department of Surgery, King's College School of Medicine, The Rayne Institute, 123 Coldharbour Lane, London SE5 9NU, UK.*

**Summary** Oestrogen receptor protein (ER) was detected in 9 of 11 samples of malignant breast tissue and 8 of 9 samples of normal breast tissue. Levels of cytosolic ER ($ER_c$) in malignant breast were 21–1102 fmol mg$^{-1}$ soluble protein (Kd $1.8 \times 10^{-9}$–$3.1 \times 10^{-8}$ mol l$^{-1}$) and those of nucleosolic ER ($ER_n$), 13–526 fmol mg$^{-1}$ soluble protein (Kd $2.1 \times 10^{-9}$–$1.4 \times 10^{-8}$ mol l$^{-1}$). In normal breast tissue $ER_c$ levels were 33–640 fmol mg$^{-1}$ soluble protein (Kd $1.3 \times 10^{-10}$–$3.2 \times 10^{-9}$ mol l$^{-1}$), $ER_n$ was detected in only 2 samples, 8 and 87 fmol mg$^{-1}$ soluble protein with Kd $3.2 \times 10^{-9}$ and $1.4 \times 10^{-9}$ l mol$^{-1}$ respectively. 17α-ethinyl-13β-ethyl-17β-hydroxy-4,15-gonadiene-3-one (gestodene), a new synthetic progestogen displaced $^3$H-oestradiol ($^3$H-$E_2$) from both $ER_c$ and $ER_n$ in malignant tissue but not in normal breast, or these receptors from endometrial tissue. In competition studies gestodehe was ∼3 times more effective in displacing $^3$H-$E_2$ from $ER_c$ and $ER_n$ in malignant breast tissue than the natural ligand. Quantitation of ER by gestodene were $ER_c$, 12–1134 fmol gestodene bound mg$^{-1}$ soluble protein (Kd $1 \times 10^{-9}$–$8.1 \times 10^{-9}$ mol l$^{-1}$); $ER_n$, 17–531 fmol gestodene bound mg$^{-1}$ soluble protein (Kd $1.6 \times 10^{-9}$–$1.1 \times 10^{-8}$ mol l$^{-1}$). L-13-ethyl-17α-ethinyl, 17β-hydroxy-gonen-3-one (levonorgestrel) showed no binding to ER in malignant breast, normal breast or endometrial tissue. In circulation both gestodene and levonorgestrel displaced $E_2$ from sex hormone binding globulin more than any of the androgens tested. These results suggest that gestodene is a progestogen with oestrogenic and/or antioestrogenic properties and provide strong evidence for differences in ER from malignant and normal breast tissue.

Oestrogen receptor (ER) in human carcinoma of the breast is the most widely studied steroid receptor. ER has been extensively purified and characterised (Jensen *et al.*, 1982) and perhaps together with the glucocorticoid receptor more is known about its physicochemical forms and characteristics than any other receptor (McGuire *et al.*, 1978; Grody *et al.*, 1982). However, many points of contention still remain (King & Green, 1984; Welshons *et al.*, 1984; Szego & Pietra, 1985).

Recently, Jasper *et al.* (1985) reported different physicochemical forms of ER in rat uterus and pituitary gland based on the hypothesis of a monomer-dimer relationship, and Brown *et al.* (1984) found that the $E_2$ dependent pS2 gene was expressed in the MCF-7 cell line and malignant breast samples but not in normal breast or ER negative cell lines. Similarly, there have been many reports on multiple receptor forms in tissues from animals of different ages and endocrine status (Jasper *et al.*, 1985), on the sedimentation behaviour of molybdate stabilised, non-activated ER, on ER bound to $E_2$ or to antioestrogens and salt or heat-activated receptor (Katzenellenbogen *et al.*, 1978, 1981; McGuire *et al.*, 1978; Grody *et al.*, 1982 and Keen *et al.*, 1984). To date, however, in no organ in any species have differences in binding of a particular steroid metabolite or analogue by ER been reported in a malignant tissue as compared to the normal tissue of the same organ.

Here we report the significant binding of a synthetic progestogen to ER in human malignant breast tissue and its total lack of binding to ER in normal breast tissue or to ER in endometrium. This is all the more surpising because the 'down regulation' of ER by progestogens has been reported (Katzenellenbogen, 1980) but the binding of this class of hormones to ER has not. As this new progestogen may form part of an oral contraceptive preparation, its binding in circulation and to specific proteins in tissues requires investigation and is reported here.

### Materials and methods

$^3$H-gestodene (specific activity 2.15 TBq mmol$^{-1}$), $^3$H-levonorgestrel (specific activity 1.44 TBq mmol$^{-1}$) and the corresponding radioinert compounds were a gift from Schering Chemicals (UK) Ltd. $^3$H-oestra-1,3,5(10)-triene-3,17β-diol (Oestradiol, $E_2$) (specific activity 3.85 TBq mmol$^{-1}$, $^3$H-5α-androstan-17β-ol-3-one (5α-dihydrotestosterone, DHT) (specific

© The Macmillan Press Ltd., 1986

activity 5.30 TBq mmol$^{-1}$), $^{3}$H-17$\beta$-hydroxyandrost-4-en-3-one (testosterone) (specific activity 3.89 TBq mmol$^{-1}$), $^{3}$H-5$\alpha$-androstan-3$\alpha$(3$\beta$)-diols (specific activities 1.52 TBq mmol$^{-1}$ each), $^{3}$H-11$\beta$,17,21-trihydroxy pregn-4-ene-3,20-dione (cortisol) (specific activity 3.0 TBq mmol$^{-1}$) and $^{3}$H-pregn-4-ene-3,20-dione (progesterone) (specific activity 3.66 TBq mmol$^{-1}$) were purchased from Amersham International, UK. Radioinert steroids were obtained from Sigma Chemical Co. UK. In all instances the purity of the steroids used was greater than 99.9% as determined by thin layer chromatography before use. Sephadex G-25, Sephadex LH-20 and Sepharose 6B were obtained from Pharmacia (GB) Ltd. Cibacron Blue 3GA-Sepharose 6B was prepared as described by Heyns and De Moor (1974). Radioactivity was determined in a Packard Tri-Carb liquid scintillation counter with an efficiency of 40% using 'Optiphase Safe' (LKB/Fisons) as scintillant. Data were analysed by Scatchard plots (Scatchard, 1949), with resolution of curvilinear plots by the method of Chamness and McGuire (1975).

Breast tissue was obtained at operation; malignant breast samples were confirmed histologically and normal breast samples were obtained either from surrounding tissue (samples 1–6, Table I) which was histologically confirmed as normal or from operative breast reduction (samples 7–9, Table II) and were stored in liquid nitrogen until assayed.

*Estimation of ER*

Cytosol and nucleosol fractions were prepared as previously described (Greenway *et al.*, 1981; Iqbal *et al.*, 1983) and the original tissue weight:volume buffer was 1:20 in the incubates. Tissue samples were manipulated below 4°C and were homogenised in TED buffer (10 mM Tris, 1.5 mM EDTA and 1.5 mM dithiothreitol, pH 7.4) using an Ultra-Turrax homogeniser before centrifugation at 160,000 *g* for 1 h, the resulting supernatant was used as cytosol. The remaining pellet was washed with TES buffer (10 mM Tris, 1 mM EDTA and 250 mM sucrose, pH 7.4), centrifuged at 800 *g* for 10 min and the supernatant discarded. The pellet was then homogenised in TSMK buffer (10 mM Tris, 250 mM sucrose, 5 mM $MgCl_2$ and 25 mM KCl, pH 7.5), centrifuged at 800 *g* for 10 min and the supernatant discarded. The pellet was washed twice with TSMK bufffer and finally suspended in TKED buffer (TED buffer containing 0.5 M KCl). The suspension was kept at 4°C for 1 h to extract nuclear receptor and then centrifuged at 15,000 *g* for 30 min, the supernatant being retained as nucleosol.

ER was measured using the two-tier column microassay employing Cibacron Blue 3GA-Sepharose 6B for the affinity immobilisation of the receptor and the steroid bound to it (Iqbal *et al.*, 1985). Aliquots (0.4 ml) of cytosol or nucleosol were incubated with a constant amount (6,000 c.p.m.) of $^{3}$H-$E_2$ and increasing amounts of radioinert $E_2$ (0–18.4 pmol). Cytosols were assayed after 2 h of incubation and nucleosols were assayed after 18 h of incubation. In the assay 0.1 ml aliquots of these incubates were applied to the microassay columns in duplicate. The column comprises a glass microcolumn fitted with a cellulose acetate plug, the upper layer consisting of 0.5 ml of Cibacron Blue 3GA-Sepharose 6B and the lower layer 1 ml of Sephadex LH-20. The columns were eluted with either 2.7 ml cytosol assay buffer (10 mM Tris, 1.5 mM EDTA, pH 7.4) for the cytosolic ER or 2.7 ml nucleosol assay buffer (10 mM Tris, 250 mM sucrose, 5 mM $MgCl_2$, 25 mM KCL, pH 7.4). After cutting the columns at the interface of the two gels, the radioactivity in the Blue gel fraction was determined. Eleven samples of malignant breast, 9 of normal breast and 3 of endometrium were assayed.

*Competition studies for ER*

In samples of normal breast ($n=9$), malignant breast ($n=11$) and endometrium ($n=3$), cytosolic and nucleosolic preparations were made as above. Aliquots (0.4 ml) of these were incubated (cytosols for 2 h and nucleosols for 18 h) with a constant amount of $^{3}$H-$E_2$ (6,000 c.p.m.) and varying amounts (0–16 pmol) of either gestodene or levonorgestrel, and also using varying amounts (0–18.4 pmol) of radioinert $E_2$. Displacement of the $^{3}$H-$E_2$ was studied with the microassay.

*Determination of ER employing gestodene as the binding ligand*

The assay for $ER_c$ and $ER_n$ were carried out on all samples of malignant, normal and endometrial tissues exactly as the ER assay described above except that a constant amount of $^{3}$H-gestodene (6,500 c.p.m.) and varying amounts (0–16 pmol) of radioinert gestodene were employed as the binding ligands. To prevent artefactual measurement of other receptors, large excesses (100 × fold) of radioinert DHT, progesterone and cortisol were included in the incubates to saturate androgen receptor, progesterone receptor and glucocorticosteroid receptor respectively.

Protein concentrations were measured using the BCA protein assay system obtained from Pierce UK Ltd. employing human serum albumin as standard.

*Competition studies in circulation*

(i) *Displacement from sex hormone binding globulin* (*SHBG*) Pooled late pregnancy serum representing an SHBG value of 250 nmol DHT bound $l^{-1}$ was diluted 1:20 in buffer (0.05 M Tris, 0.005 M $CaCl_2$, pH 7.5) and was used throughout. Aliquots (0.4 ml) of the above dilution were incubated as described for the two-tier column SHBG assay (Iqbal & Johnson, 1977) with constant amounts (25,000 c.p.m.) of either $^3$H-DHT, $^3$H-testosterone, $^3H$-$E_2$, $^3$H-5$\alpha$-androstane-3$\alpha$(or 3$\beta$),17$\beta$-diol and varying amounts (0–180 pmol) of their respective radioinert moieties. In a parallel series of experiments displacement of the above tritiated steroids was carried out with 0–165 pmol amounts of either radioinert gestodene or radioinert levonorgestrel.

(ii) *Displacement from corticosteroid binding globulin* (*CBG*) The two-tier columns were prepared as above except that the Sephadex LH-20 gel in the lower tier was replaced by 1 ml of Sephadex G-25. The rest of the experimental conditions were as in (i) above. Displacement of $^3$H-progesterone and $^3$H-cortisol was studied by using varying concentrations (0–180 pmol) of their respective radioinert ligands and in a parallel series of experiments. displacement of $^3$H-progesterone and $^3$H-cortisol was studied by using varying amounts of radioinert gestodene or levonorgestrel (0–165 pmol).

### Results

Of the 11 samples of the carcinoma of the breast assayed 9 were $ER_c$ positive (1–6, 8, 10 and 11, Table I) and 8 were $ER_c$ and $ER_n$ positive (1–3, 5, 8, 10 and 11) employing $E_2$ as the binding ligand (Table I, Figure 1). In normal breast obtained from the corresponding carcinoma of the breast tissue (1–6, Table I) $ER_c$ was positive in samples 1, 3–6 (Table II); $ER_c$ in samples 7–9 was also positive (Table II, Figure 2). $ER_n$ in normal breast was detected only in two samples (3 and 8, Table II). All three samples of endometrium were $ER_c$ and $ER_n$ positive, $ER_c$: 191, 495, 326 fmol $E_2$ bound $mg^{-1}$ soluble protein and $ER_n$: 34, 176 and 59 fmol $mg^{-1}$ soluble protein respectively (Kd 7.1, 6.8 and $7.2 \times 10^{-9}$ mol $l^{-1}$ for $ER_c$, and for $ER_n$ 3.0, 3.5 and $4.6 \times 10^{-9}$ mol $l^{-1}$ respectively).

Competition studies showed that gestodene displaced $^3H$-$E_2$ from $ER_c$ and $ER_n$ in malignant

**Table I** Oestrogen receptor in cytosol ($ER_c$) and nucleosol ($ER_n$) assayed in carcinoma of the breast tissue using oestradiol ($E_2$) and gestodene as ligands respectively. Kd $\times 10^{-9}$ unless otherwise stated

| | *$ER_c$* | *$ER_n$* | *$ER_c$* | *$ER_n$* |
|---|---|---|---|---|
| *Sample no.* | *fmol $E_2$ bound $mg^{-1}$ soluble protein* | | *fmol gestodene bound $mg^{-1}$ soluble protein* | |
| 1 | 505 (Kd 7.7) | 186 (Kd 3.2) | 1134 (Kd 8.0) | Not measured |
| 2 | 21 (Kd 1.8) | 500 (Kd 2.1) | 12 (Kd 1.0) | 531 (Kd 1.6) |
| 3 | 1102 (Kd $3.1 \times 10^{-8}$) | 526 (Kd $1.4 \times 10^{-8}$) | 484 (Kd 8.1) | 526 (Kd $1.1 \times 10^{-8}$) |
| 4 | 625 (Kd 8.9) | Negative | 625 (Kd 5.5) | Negative |
| 5 | 195 (Kd 4.8) | 13 (Kd 1.7) | 109 (Kd 3.8) | 17 (Kd 1.6) |
| 6 | 45 (Kd 3.7) | Negative | 79 (Kd 3.8) | Negative |
| 7 | Negative | Negative | Negative | Negative |
| 8 | 106 (Kd 3.1) | 49 (Kd 3.6) | 126 (Kd 3.4) | 57 (Kd 4.4) |
| 9 | Negative | Negative | Negative | Negative |
| 10 | 23 (Kd 2.7) | 192 (Kd 2.2) | 36 (Kd 2.0) | 216 (Kd 1.9) |
| 11 | 111 (Kd 2.1) | 52 (Kd 3.2) | 93 (Kd 1.8) | 39 (Kd 1.0) |

**Table II** Oestrogen receptor in cytosol ($ER_c$) and nucleosol ($ER_n$) assayed in normal breast tissue. $Kd \times 10^{-9} mol l^{-1}$ unless otherwise stated

| *Sample No.* | $ER_c$ | $ER_n$ |
|---|---|---|
| | *fmol oestradiol bound mg$^{-1}$ soluble protein* | |
| 1 | 640 (Kd $1.3 \times 10^{-10}$) | Negative |
| 2 | Negative | Negative |
| 3 | 233 (Kd 1.5) | 87 (Kd 1.4) |
| 4 | 322 (Kd 1.5) | Negative |
| 5 | 166 (Kd $8.8 \times 10^{-10}$) | Negative |
| 6 | 63 (Kd 3.2) | Negative |
| 7 | 43 (Kd 3.2) | Negative |
| 8 | 33 (Kd 3.5) | 8 (Kd 3.1) |
| 9 | 105 (Kd 2.6) | Negative |

breast tissue samples by a factor 3 times greater than the natural ligand (Figure 3), 50% displacement of $E_2$ being caused by $7 \times 10^{-4}$ nmol $E_2$ added as compared to $2.2 \times 10^{-4}$ nmol gestodene added. No displacement of $^3H$-$E_2$ was observed by gestodene in any sample of normal breast nor the 3 samples of endometrium either from $ER_c$ or $ER_n$. Levonorgestrel showed no displacement of $^3H$-$E_2$ from $ER_c$ or $ER_n$ obtained from any sample of either malignant or normal breast (Figures 1 and 2) or endometrial tissue.

When the other receptors had been saturated with excess of their natural ligands ER measured by gestodene showed values comparable to those obtained when the natural ligand had been employed (Table I) with approximately similar Kd values. $ER_c$ or $ER_n$ observed to be negative using $E_2$ as the ligand were also found to be negative using gestodene as the ligand (Table I). Linear regression analysis carried out between $ER_c$ measured with $E_2$ and $ER_c$ measured with gestodene gave an $r$ value of $+0.6901$ (not significant). Similar analysis for $ER_n$ gave an $r$ value of $+0.9987$ ($P<0.01$).

Fifty percent displacement of $^3H$-DHT, $^3H$-testosterone, $^3H$-$E_2$, $^3H$-5$\alpha$-androstane-3$\alpha$,17$\beta$-diol and $^3H$-5$\alpha$-androstane-3$\beta$,17$\beta$-diol from SHBG in circulation by gestodene were achieved by concentrations 560%, 235%, 65%, 75% and 80% of their natural ligand respectively. Similar studies with levonorgestrel showed that gestodene was ~20% more effective in these displacements than was the former analogue. No displacement of $^3H$-progesterone or $^3H$-cortisol from CBG in circulation was caused by either gestodene or levonorgestrel.

**Figure 1** Scatchard plots of binding of oestradiol O——O, gestodene △——△, and levonorgestrel ×——× in (a) cytosol, and (b) nucleosol of carcinoma of the breast tissue.

**Figure 2** Scatchard plots of binding of oestradiol ○——○, gestodene △——△, and levonorgestrel ×——× in (a) cytosol, and (b) nucleosol of normal breast tissue.

**Figure 3** Semi-logarithmic plot of % displacement of $^{3}$H-oestradiol by radioinert oestradiol ○——○, and gestodene ×——× in malignant breast cytosols. Mean ±s.d. of 7 determinations.

### Discussion

Gestodene is structurally closely related to levonorgestrel and its optical isomer d-norgestrel. The latter compound has been shown to displace sex-steroids from SHBG in circulation (Victor *et al.*, 1976). While it is surprising that a progestogen should not displace progesterone from CBG in serum, the displacement of sex-steroids from SHBG may be related to the close structural similarities of the above compounds which all possess a hydroxyl group in the 17$\beta$ position of the D ring on the steroid molecule (Figure 4). The clinical implications of this displacement of sex steroids in circulation area change in the balance of free sex-steroids and have been discussed by the above authors.

**Figure 4** Structures of oestra, 1,3,5(10)-triene-3,17$\beta$-diol (oestradiol), pregn-4-ene-3,20-dione (progesterone), 17$\alpha$-ethinyl-13$\beta$-ethyl-17$\beta$-hydroxy-4,15-gonadiene-3-one (gestodene), and L-13-ethyl-17$\alpha$-ethinyl-17$\beta$-hydroxy-gonen-3-one (levonorgestrel).

In relation to the binding of gestodene to ER in malignant breast tissue, the findings are much more unexpected and have far reaching implications. This study demonstrates for the first time that a steroidal compound exhibits binding to ER from malignant breast but not to that from normal breast. This indicates a structural difference between the two receptors. In competition studies the results show that gestodene can displace $^3H\text{-}E_2$ by about 3-fold as compared to the natural ligand, however, when ER from malignant breast tissue is measured using this synthetic steroid there is little difference in the total steroid bound or in the dissociation constant suggesting that gestodene prevents binding of $E_2$ not only by competing for the binding site on ER but perhaps also by interfering with the formation of $E_2$–ER complex. The high positive correlation between $ER_n$ measured with $E_2$ and that measured with gestodene supports this hypothesis.

The evidence presented here suggests that the binding site and therefore the structure of ER extracted from malignant breast is different from that in either normal breast of endometrium and that gestodene may be of clinical value as an antioestrogen in the management of malignant breast disease.

The financial assistance of Schering Chemicals (UK.) Ltd. is gratefully acknowledged.

### References

BROWN, A.M.C., JELTSCH, J., ROBERTS, M. & CHAMBON, P. (1984). Activation of pS2 gene transcription is a primary response to estrogen in the human cancer cell line MCF-7. *Proc. Natl Acad. Sci., USA*, **81**, 6344.

CHAMNESS, G.C. & McGUIRE, W.L. (1975). Scatchard plots, common errors in correction and interpretation. *Steroids*, **26**, 538.

GREENWAY, B.A., IQBAL, M.J., JOHNSON, P.J. & WILLIAMS, R. (1981). Oestrogen receptor proteins in malignant and fetal pancreas. *Br. Med. J.*, **283**, 751.

GRODY, W.W., SCHRADER, W.T. & O'MALLEY, B.W. (1982). Activation, transformation and subunit structure of steroid hormone receptors. *Endocrine Rev.*, **3**, 141.

HEYNS & DE MOOR (1974).

IQBAL, M.J. & JOHNSON, M.W. (1977). Study of steroid protein binding by a novel 'two-tier' column employing Cibacron Blue F3G A-Sepharose 4B. I – Sex hormone binding globulin. *J. Steroid Biochem.*, **8**, 977.

IQBAL, M.J., WILKINSON, M.L., JOHNSON, P.J. & WILLIAMS, R. (1983). Sex steroid receptor proteins in foetal, adult and malignant liver tissue. *Br. J. Cancer*, **48**, 791.

IQBAL, M.J., CORBISHLEY, T.P., WILKINSON, M.L. & WILLIAMS, R. (1985). A microassay for the determination of binding parameters of oestrogen and androgen receptors employing Cibacron Blue 3GA-Sepharose 6B. *Analyt. Biochem.*, **144**, 79.

JASPER, T.W., RUH, M.F. & RUH, T.S. (1985). Estrogen and antiestrogen binding to rat uterine and pituitary estrogen receptor: Evidence for at least two physico-chemical forms of the estrogen receptor. *J. Steroid Biochem.*, **23**, 537.

JENSEN, E.V., GREENE, G.L., CLOSS, L.E., DE SOMBRE, E.R. & NADJI, M. (1982). Receptors reconsidered: A 20-year perspective. In *Recent Progress in Hormone Research*, Greep, R.O. (ed) p. 17. Academic Press: New York.

KATZENELLENBOGEN, B.S., KATZENELLENBOGEN, J.A., FERGUSON, E.R. & KRAUTHAMMER, N. (1978). Antiestrogen interaction with uterine estrogen receptor. *J. Biol. Chem.*, **253**, 697.

KATZENELLENBOGEN, B.S. (1980). Dynamics of steroid hormone receptor interaction. *Ann. Rev. Physiol.*, **42**, 17.

KATZENELLENBOGEN, B.S., PAVLICK, E.J., ROBERTSON, D.W. & KATZENELLENBOGEN, J.A. (1981). Interaction of a high affinity antiestrogen (α-[4-pyrrolidinoethoxy]phenyl-4-hydroxy-α-nitrostilbene (CI628M) with uterine estrogen receptor. *J. Biol. Chem.*, **256**, 2908.

KEEN, J.L., SWEET, F., RUH, M.F. & RUH, T.S. (1984). Interaction of the radiolabelled high-affinity anti-oestrogen [$^3$H] H1285 with the cytoplasmic oestrogen receptor. *Biochem. J.*, **217**, 819.

KING, W.J. & GREEN, G.L. (1984). Monoclonal antibodies localize oestrogen receptor in the nuclei of target cells. *Nature*, **307**, 745.

McGUIRE, W.L., ZAVA, D.T., HORWITZ, K.B., GAROLA, R.E. & CHAMNESS, G.C. (1978). Receptors and breast cancer: Do we know it all? *J. Steroid Biochem.*, **9**, 461.

SCATCHARD, G. (1949). The attraction of proteins for small molecules and ions. *Ann. N.Y. Acad. Sci.*, **51**, 660.

SZEGO, C.M. & PIETRAS, R.J. (1985). Subcellular distribution of oestrogen receptors. *Nature*, **317**, 88.

VICTOR, A., WEINER, E. & JOHANSSON, E.D.B. (1976). Sex hormone binding globulin: The carrier protein for d-norgestrel. *J. Clin. Endocrinol. Metab.*, **43**, 244.

WELSHONS, W.V., LIEBERMAN, M.E. & GORSKI, J. (1984). Nuclear localization of unoccupied oestrogen receptors. *Nature*, **307**, 747.